# Association of homocysteine and polymorphism of methylenetetrahydrofolate reductase with early-onset post stroke depression

**DOI:** 10.3389/fnut.2022.1078281

**Published:** 2022-12-06

**Authors:** Jingyuan Zhang, Chang Zeng, Xia Huang, Qiao Liao, Hengshu Chen, Fan Liu, Dongren Sun, Shihang Luo, Yeqing Xiao, Weiye Xu, Danfeng Zeng, Mingyu Song, Fafa Tian

**Affiliations:** ^1^Department of Neurology, Xiangya Hospital, Central South University, Changsha, China; ^2^National Clinical Research Center for Geriatric Disorders, Xiangya Hospital, Central South University, Changsha, China; ^3^Health Management Center, Xiangya Hospital, Central South University, Changsha, China; ^4^Department of Critical Care Medicine, The First People’s Hospital of Huaihua, Huaihua, China; ^5^Department of Neurology, Hengyang Central Hospital, Hengyang, China; ^6^Department of Human Anatomy and Neurobiology, School of Basic Medicine, Central South University, Changsha, China; ^7^Department of Neurology, Xiangtan Central Hospital, Xiangtan, China

**Keywords:** post stroke depression, homocysteine, single nucleotide polymorphisms, MTHFR, MTRR, MTR

## Abstract

**Background:**

Homocysteine (Hcy) has been indicated to be involved in pathophysiology of post stroke depression (PSD). There is a lack of research to study the relationship between Hcy metabolism genes and PSD. Our study aims to investigate the relationship among Hcy metabolism genes, Hcy, and early-onset PSD.

**Materials and methods:**

We recruited 212 patients with stroke and collected their peripheral blood sample, clinical data, and laboratory test on admission. 12 single nucleotide polymorphisms (SNPs) in methylenetetrahydrofolate reductase (MTHFR), methionine synthase reductase (MTRR), and methionine synthase (MTR) genes were genotyped by high-resolution melt analysis. PSD was diagnosed by DSM-V at 2 weeks after stroke. Binary logistic regression and haplotype analysis were used to examine the association between Hcy metabolism genes and PSD. Mediation analysis was performed to clarify whether the SNPs exerted their effect on PSD by affecting the Hcy level.

**Results:**

81 patients were diagnosed with PSD, and the incidence rate was 38.2%. Hcy level in PSD group was significantly higher than it in non-PSD group (*p* = 0.019). MTHFR rs1801133 AA genotype an A allele were associated with an elevated risk of PSD after adjustment for some confounding factors (OR = 4.021, 95% CI: 1.459∼11.080, *p* = 0.007 for AA genotype; OR = 1.808, 95% CI: 1.172∼2.788, *p* = 0.007 for A allele). Furthermore, the effect of MTHFR rs1801133 AA genotype on PSD was mediated by Hcy (OR = 1.569, 95% CI: 0.013∼3.350, *p* < 0.05).

**Conclusion:**

MTHFR rs1801133 and Hcy were associated with PSD, and MTHFR rs1801133 may exert an effect on PSD *via* mediating Hcy level. This offers a new perspective for treating PSD and understanding the mechanism of PSD.

## Introduction

Post stroke depression (PSD) is a common neuropsychiatric sequela after stroke with an incidence rate of approximately 30% at any time after stroke (1, 2). PSD is associated with a poor prognosis of stroke in the form of worse functional and motor recovery, poorer cognitive impairment, lower quality of life, and higher mortality (1, 3, 4). It is of substantial clinical significance to achieve early identification, accurate diagnosis, and timely treatment of PSD. However, due to the paucity of reliable objective biomarkers of PSD, as well as the impediment of stroke-related neurological symptoms such as aphasia and abulia, many patients with PSD cannot be promptly diagnosed and treated (5). Therefore, investigating the mechanism of PSD is of great urgency for the early identification and treatment of PSD.

Homocysteine (Hcy), a sulfur-containing amino acid, is an essential intermediate product during the metabolism of methionine to cysteine. Previous researches have proved that elevated serum Hcy level was a risk factor for ischemic stroke and could predict mortality from ischemic stroke, especially in the large-artery atherosclerosis subtype (6, 7). Folic acid supplementation can effectively lower the risk and improve the outcome of stroke (8, 9). Meanwhile, a meta-analysis showed that the Hcy level was higher in depressed subjects compared with healthy controls, and elevated Hcy was associated with lifetime major depressive disorder and currently experiencing depressive symptoms (10–12). Lowering Hcy by promoting vitamin B12 and folate-rich food may help ameliorate depression and anxiety (13). Notably, a previous study reported elevated Hcy at admission was related to PSD at 3 months after stroke (14). High sensitivity C-reactive protein combined with Hcy could predict PSD at 3 months and 1 year after stroke more accurately, compared to any single factor (15, 16). Therefore, we speculate Hcy may be closely related to the occurrence and development of PSD. However, the mechanism behind the relationship between Hcy and PSD remains unknown.

Homocysteine (Hcy) concentration in serum can be influenced by diet, renal function, and genetic factors (17). Several genes, including methylenetetrahydrofolate reductase (MTHFR), methionine synthase (MTR), and methionine synthase reductase (MTRR), take part in the Hcy remethylation pathway *via* encoding related enzymes (18–20). MTHFR catalyzes the conversion of 5,10-methylene-tetrahydrofolate to 5-methyl-tetrahydrofolate, which serves as the substrate for Hcy into methionine catalyzed by MTR (21, 22). In this reaction, MTR require cobalamin(I) as a coenzyme, and cobalamin(I) is rapidly oxidized to cobalamin(II), resulting in the inactivation of MTR. MTRR maintains the activity of MTR by reductive methylation of cobalamin(II) (20). Little efforts on the role of Hcy metabolism genes in PSD have been made. Mei et al. only focus on the association between rs1801133 (MTHFR) and PSD and found that rs1801133 AG genotype and A allele increased the risk of PSD (23). However, whether other polymorphisms of Hcy metabolism genes are linked to PSD needs further investigation.

This study is aimed to examine the change in Hcy level in patients with PSD at 2 weeks after stroke (early-onset PSD). Furthermore, we explored the polymorphisms of Hcy metabolism genes to reveal the mechanism of altered Hcy levels in PSD.

## Materials and methods

### Study population

The patient population comprised 227 acute ischemic stroke patients consecutively admitted to Xiangya Hospital Central South University, Changsha from July 2019 to August 2021. The Ethics Committee of Xiangya Hospital, Central South University approved the protocol (No. 201910842). All patients provided written informed consent in accordance with the Declaration of Helsinki and its later amendments. The inclusion criteria were as follows: (1) diagnosed stroke patients by neurological examination, cerebral computed tomographic scan, and magnetic resonance imaging; (2) age between 18 and 75 years old; and (3) hospital admission within 2 weeks after stroke onset. The exclusive criteria were as follows: (1) unable to complete the evaluations and questionnaires because of disturbance of consciousness and comprehension, visual and hearing disturbance, and communication problems caused by aphasia and dysarthria; (2) comorbid with other concomitant neuropsychiatric diseases, for example, Parkinson’s disease, Alzheimer’s disease, and epilepsy; (3) brain dysfunction caused by other non-vascular causes, for instance, brain trauma, brain tumor, encephalitis and so on; (4) history of depression and other mental disorder.

### Data collection and clinical assessment

We collected information on demographics, medical history, and clinical characteristics. The National Institutes of Health Stroke Scale (NIHSS) was used to evaluate stroke severity within 24 h after hospital admission by neurologists. Barthel Index (BI) scores and modified Rankin Scale (mRS) scores were also collected to assess the activity of daily living. Mini-mental State Examination (MMSE) was used to measure cognitive function. Diagnostic and Statistical Manual of Mental Disorders, 5th edition (DSM-V) was adopted to diagnose PSD by a psychiatrist at 2 weeks after stroke. The severity of depressive and anxious symptoms was evaluated with the Hamilton depression scale 17 items (HAMD-17) and Hamilton anxiety scale (HAMA) respectively. All researchers involved in conducting clinical assessments were blinded to genetic and serum biochemical data after uniform training.

### Blood sample collection, storage, and measurements

We collected peripheral blood samples of patients on admission in vacutainer tubes containing the anticoagulant ethylenediaminetetraacetic acid and stored them at −80°C until use. The concentration of plasma Hcy was measured using the kit (Maccura Biotechnology Co., Ltd., Chengdu, China) according to the manufacturer’s instruction in the clinical laboratory of Xiangya Hospital.

### Single nucleotide polymorphism selection and genotyping

According to information in the National Center for Biotechnology Information SNP database,^1^ 12 SNPs of MTHFR, MTR, and MTRR were selected minor allele frequency of > 5% and previously reported (24–26). All SNPs were genotyped using a SNPscan™ multiplex SNP typing Kit (Cat#: G0104K, Genesky Biotechnologies Inc., Shanghai, China). The SNPscan assay technique is a rapid multiplex genetic screening system, and the basic principle of this technology is based on double ligation and multiplex fluorescent polymerase chain reaction (PCR). The procedure of this technique has been reported in the previous study (27). For quality control, 5% of duplicate DNA samples were analyzed with the same process.

### Statistical analysis

We conducted statistical analysis by SPSS version 13.0 for windows (SPSS Inc., Chicago, IL, USA). Mann-Whitney test, Wilcoxon signed rank test, and Student’s *t*-test were used to determine whether the differences of median existed. Differences between proportions were assessed with the χ2-test or Fisher exact test. *F* test was assessed for equal variance, and normality was assessed by the Shapiro-Wilk normality test. Binary logistic regression analysis was adopted to examine the association between genotypes and PSD. The statistical significance threshold was set to a two-sided *p*-value < 0.05. The bootstrapping test was performed using the SPSS PROCESS version 4.1 to test the statistical significance of the mediating effect. The significance of the mediation effect was conducted using 5,000 bootstrapped iterations. Hardy-Weinberg equilibrium (HWE) test, linkage disequilibrium (LD), and haplotype association analysis were conducted by the SHEsis, an online program^2^ (28, 29). The results of categorical variables were expressed as percentages. Continuous variables were shown as the median and interquartile range (IQR) or the mean ± standard deviation (SD) depending on the normal or non-normal distribution of the data.

## Results

### Characteristics of the study population

**FIGURE 1:**
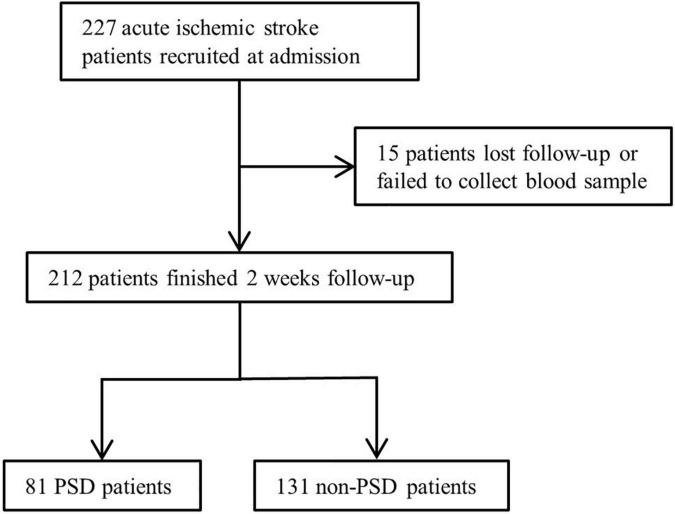
Study recruitment profile. PSD, post stroke depression.

Due to data missing and loss follow-up, 15 patients were excluded (Figure 1). We included 212 patients with ischemic stroke, and 81 patients were diagnosed with PSD, for a percentage of 38.2%. The demographic and clinical characteristics are shown in Table 1. Compared to the non-PSD group, the PSD group showed a higher NIHSS score [4 (2, 8.5) vs. 2 (1, 4), *p* < 0.001], mRS score [3 (1, 4) vs. 2 (1, 3); *p* < 0.001], HAMD score [14 (11, 20) vs. 5 (2, 8), *p* < 0.001], HAMA score [14 (9.5, 19) vs. 5.5 (3, 8.75), *p* < 0.001], and lower BI score [75 (35, 100) vs. 100 (70, 100); *p* < 0.001]. There were no significant differences between stroke patients with and without PSD in age, gender, marital status, educational level, body mass index, vascular risk factors (smoking, drinking, hypertension, and diabetes mellitus) location of lesion TOAST classification, MMSE, and serum biochemical index (creatinine, total cholesterol, triglycerides, low-density lipoprotein cholesterol, high-density lipoprotein cholesterol).

**TABLE 1:** Clinical and demographic characteristics of post stroke depression (PSD) and non-PSD patients.

Characteristics	PSD (*n* = 81)	Non-PSD (*n* = 131)	*p*-value
Age (years), mean ± SD	57.91 ± 9.66	56.73 ± 11.65	0.443
Male, n (%)	54 (66.7)	93 (71.0)	0.507
Married, n (%)	77 (95.1)	119 (90.8)	0.258
Educational level			
Junior middle school and below, n (%)	46 (56.8)	66 (50.4)	0.634
Senior high/polytechnic school, n (%)	21 (25.9)	37 (28.2)	
University and above, n (%)	14 (17.3)	28 (21.4)	
BMI (kg/m^2^), mean ± SD	23.76 ± 2.93	23.70 ± 2.94	0.881
History of smoking, n (%)	42 (51.9)	76 (58.0)	0.380
History of drinking, n (%)	39 (48.1)	69 (52.7)	0.522
History of hypertension, n (%)	61 (75.3)	87 (66.4)	0.170
History of diabetes, n (%)	30 (37.0)	33 (25.2)	0.067
Lesion location			
Anterior circulation, n (%)	59 (72.8)	81 (61.8)	0.170
Posterior circulation, n (%)	18 (22.2)	45 (34.4)	
Both, n (%)	4 (4.9)	5 (3.8)	
TOAST classification			
Large-artery atherosclerosis, n (%)	60 (74.1)	95 (78.5)	
Cardioembolism, n (%)	10 (12.3)	13 (10.7)	
Small-vessel occlusion, n (%)	5 (6.2)	7 (5.8)	0.832
Stroke of other determined etiology, n (%)	2 (2.5)	5 (4.1)	
Stroke of undetermined etiology, n (%)	4 (4.9)	11 (9.1)	
NIHSS score, median (IQR)	4 (2, 8.5)	2 (1, 4)	< 0.001
BIscore, median (IQR)	75 (35, 100)	100 (70, 100)	< 0.001
mRS score, median (IQR)	3 (1, 4)	2 (1, 3)	< 0.001
MMSE score, median (IQR)	25 (22, 27.5)	26 (23, 29)	0.122
HAMD-17 score, median (IQR)	14 (11, 20)	5 (2, 8)	< 0.001
HAMA score, median (IQR)	14 (9.5, 19)	5.5 (3, 8.75)	< 0.001
Cr (μmol/L), mean ± SD	80.54 ± 19.87	86.54 ± 37.44	0.185
TC (mmol/L), mean ± SD	4.90 ± 1.16	4.60 ± 1.40	0.115
TG (mmol/L), mean ± SD	2.17 ± 1.69	2.09 ± 1.48	0.704
LDL-C (mmol/L), mean ± SD	1.08 ± 0.29	1.04 ± 0.28	0.286
HDL-C (mmol/L), mean ± SD	3.11 ± 0.79	2.89 ± 1.00	0.094
Hcy (μmol/L), mean ± SD	17.06 ± 8.35	14.63 ± 6.57	0.019

Values are shown as number (percentage) or as medians (IQR) and mean (SD). Statistical significance was accepted at *p* < 0.05. PSD, post-stroke depression; BMI, body mass index; NIHSS, national institutes of health and stroke scale; BI, barthel index; mRS, modified rankin scale; MMSE, Mini-mental state examination; HAMD, hamilton depression scale; HAMA, Hamilton anxiety scale; Cr, creatinine; TC, total cholesterol; TG, triglycerides; LDL-C, low-density lipoprotein cholesterol; HDL-C, high-density lipoprotein cholesterol; Hcy, homocysteine.

### Association between genotypes of MTHFR, MTRR, MTR, Hcy, and early-onset PSD

**FIGURE 2:**
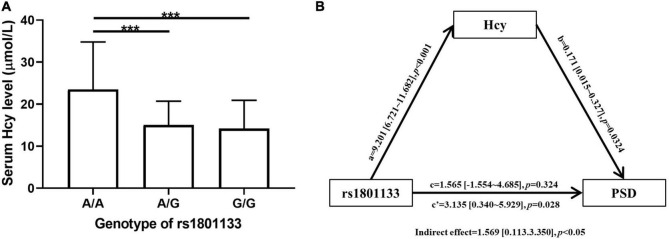
**(A)** Comparison of Homocysteine (Hcy) level among AA, AG, and GG genotype of rs1801133. Statistical significance was accepted at *p* < 0.05. ***p < 0.001. **(B)** Mediation analysis of association between rs1801133 [methylenetetrahydrofolate reductase (MTHFR)] and post stroke depression (PSD). The genotypes of rs1801133 were divided into AA and GA + GG group. The severity of PSD was represented as HAMD-17 score. (a) Direct effect of rs1801133 on Hcy; (b) Direct effect of Hcy on PSD; (c’) Total effect of rs1801133 on PSD; (c) Direct effect of rs1801133 on PSD.

As shown in Table 1, the PSD group had a higher Hcy level than non-PSD group (17.06 ± 8.35 vs. 14.63 ± 6.57, *p* = 0.019). All SNPs distribution of MTHFR, MTRR, and MTR were consistent with Hardy-Weinberg equilibrium (*p* > 0.05). The rs1801133 (MTHFR) was significantly associated with PSD. AA genotype (OR = 3.531, 95% CI: 1.351∼9.227, *p* = 0.010) and A allele (OR = 1.792, 95% CI: 1.186∼2.706, *p* = 0.006) were risk factors of PSD, and AG genotype was marginally associated with PSD (OR = 1.790, 95% CI: 0.980∼3.269, *p* = 0.058). The rs1801131 T allele was related to an elevated risk of PSD [0.573 (0.341∼0.964), *p* = 0.036]. When adjusting for confounding factors (age, gender, body mass index, NIHSS score, and MMSE score), rs1801133 AA genotype (OR = 4.021, 95% CI: 1.459∼11.080, *p* = 0.007) and A allele (OR = 1.808, 95% CI: 1.172∼2.788, *p* = 0.007) were still strongly related to PSD (Table 2), but not rs1801131 T allele. Other SNPs did not show statistically significant differences between PSD group and non-PSD group (Supplementary Table 1). In addition, the mean Hcy level concentrations according to corresponding genotypes of rs1801133 were shown in Figure 2A. The Hcy level was significantly correlated with rs1801133 (*p* < 0.001) but no other SNPs.

**TABLE 2:** Association of genotypes and alleles of methylenetetrahydrofolate reductase (MTHFR) and post stroke depression (PSD).

Genotypes and alleles	PSD	Non-PSD	OR [CI_95%_]	*p*-value	Adjusted OR [CI_95%_]	*p*-value
**rs11559040 (MTHFR)**						
G/G	72	109	Ref	–	Ref	–
G/A	9	19	0.717 [0.307∼1.673]	0.442	0.708 [0.289∼1.736]	0.451
A/A	0	3	–	0.999	–	0.999
G	153	237	0.558 [0.253∼1.227]	0.147	0.548 [0.241∼1.246]	0.151
A	9	25				
**rs1801131 (MTHFR)**						
T/T	59	77	Ref	–	Ref	–
T/G	20	47	0.555 [0.298∼1.036]	0.064	0.559 [0.290∼1.076]	0.082
G/G	2	7	0.373 [0.075∼1.861]	0.229	0.446 [0.085∼2.334]	0.339
T	138	201	0.573 [0.341∼0.964]	0.036	0.598 [0.349∼1.024]	0.061
G	24	61				
**rs1801133 (MTHFR)**						
G/G	27	66	Ref	–	Ref	–
G/A	41	56	1.790 [0.980∼3.269]	0.058	1.667 [0.883∼3.145]	0.115
A/A	13	9	3.531 [1.351∼9.227]	0.010	4.021 [1.459∼11.080]	0.007
G	95	188	1.792 [1.186∼2.706]	0.006	1.808 [1.172∼2.788]	0.007
A	67	74				
**rs20666462 (MTHFR)**						
G/G	67	98	Ref	–	Ref	–
G/A	13	32	0.594 [0.291∼1.215]	0.154	0.601 [0.285∼1.268]	0.181
A/A	1	1	1.463 [0.090∼23.794]	0.789	2.540 [0.138∼46.799]	0.531
G	147	228	0.684 [0.360∼1.300]	0.247	0.723 [0.371∼1.408]	0.340
A	15	34				

SNP, single nucleotide polymorphism; PSD, post stroke depression; OR, odds ratio; CI, confidence interval; MTHFR, methylenetetrahydrofolate reductase; Adjusted model: Adjusted for age, gender, body mass index, NIHSS score and MMSE score.

### Association between haplotype of MTHFR, MTRR, and MTR and early-onset PSD

Haplotype analysis is a powerful strategy to examine whether SNPs have a greater effect when analyzed together. As shown in Table 3, rs11559040, rs1801131, rs1801133, and rs2066462 exhibited strong LD (*D*’ > 0.95) (30). They formed four main haplotypes (AGGG, GGGA, GTAG GTGG), and those with frequency < 0.03 were excluded from the analysis (Table 4). We found that the GTAG haplotype was associated with an increased risk of PSD (OR = 1.852, 95% CI: 1.223∼2.805, *p* = 0.003), while haplotypes of MTRR and MTR were not.

**TABLE 3:** Linkage disequilibrium among rs11559040, rs1801131, rs1801133, and rs2066462.

D’/R^2^	rs11559040	rs1801131	rs1801133	rs2066462
rs11559040		0.959	0.999	0.998
rs1801131	0.319		1.000	0.972
rs1801133	0.043	0.125		0.999
rs2066462	0.011	0.492	0.065	

The *D*’ values are shown above the diagonal, and the *R*^2^ values are shown below the diagonal.

**TABLE 4:** Haplotype analysis of rs11559040-rs1801131-rs1801133-rs2066462.

Haplotype	PSD (freq)	Non-PSD (freq)	*p*-value	OR [CI_95%_]
AGGG	7.98 (0.049)	25.00 (0.095)	0.094	0.500 [0.220∼1.139]
GGGA	13.98 (0.086)	33.99 (0.130)	0.190	0.646 [0.335∼1.246]
GTAG	67.00 (0.414)	73.98 (0.282)	0.003	1.852 [1.223∼2.805]
GTGG	68.97 (0.426)	127.02 (0.485)	0.303	0.812 [0.545∼1.208]
Global result	−	−	0.015	–

OR, odds ratio; CI, confidence interval. Haplotype frequency < 0.03 in both control and case has been excluded.

### Mediation analysis

To clarify the relationship among rs1801133, Hcy, and PSD, we adopted mediation analysis to test whether rs1801133 AA genotype would affect the incidence of PSD *via* mediating the Hcy level with age, gender, body mass index, NIHSS score, and MMSE score as covariate (Figure 2B). We found that rs1801133 AA genotype was significantly associated with PSD (total effect, OR = 3.135, 95% CI: 0.340∼5.929, *p* = 0.028). However, rs1801133 AA genotype was not linked to increased risk of PSD when removing the effect of Hcy (direct effect, OR = 1.565, 95% CI: −1.554∼4.685, *p* = 0.324), meanwhile, the effect of rs1801133 on PSD was mediated *via* Hcy (indirect effect, OR = 1.569, 95% CI: 0.113∼3.350, *p* < 0.05).

## Discussion

This is the first study to determine the association among early-onset PSD, Hcy, and polymorphism of Hcy metabolism genes with a prospective cohort. We demonstrated that rs1801133 AA genotype, A allele, and haplotype GTAG (rs11559040-rs1801131-rs1801133-rs2066462) were associated with PSD, meanwhile, PSD group showed a higher Hcy level than non-PSD group. Furthermore, rs1801133 influenced PSD through mediating the Hcy levels.

In our study, 38.2% of the patients were diagnosed with PSD at 2 weeks after stroke onset. Similar to our results, a meta-analysis reported that approximately 31% of stroke survivors were found to have depression at any time-point up to 5 years after stroke (2). We discovered that PSD group showed higher NIHSS score and mRS score and lower BI score, compared to non-PSD group, which indicated the higher severity of disability and stroke in PSD group. Adverse physical conditions may be stressors of developing psychological problems like depression (31). This can explain the cause of PSD to some extent.

Methylenetetrahydrofolate reductase (MTHFR) is an essential enzyme in metabolizing folate. The rs1801133 (also named C677T) is a non-synonymous variant, leading to an amino acid change from alanine to valine. This substitution could reduce the activity of MTHFR and folate levels and elevate serum Hcy levels (19, 32). MTHFR rs1801133 mutations and folate deficiency could increase the risk of coronary heart disease and ischemic stroke in later life (33–36). Folic acid supplementation could prevent birth defects, including neural tube, abdominal wall, and congenital heart defects (37–39). The MTHFR activity of heterozygous and homozygous mutant individuals are respectively 67 and 25% of the wild-type ones (40). Consistently, we found that the Hcy level of stroke patients with rs1801133 AA genotype was higher than those with AG or GG genotype. Our study demonstrated that MTHFR rs1801133 AA genotype and A allele were closely related to early-onset PSD. Mei et al. discovered that mutations of MTHFR rs1801133 AG genotype and A allele were significantly related to the elevated risk of PSD (23), which was partially consistent with our results. The discrepancy may be attributed to the difference in assessment time of PSD and study population like age, gender composition, and so on.

We found that Hcy was significantly related to PSD. Michaela et al. discovered Hcy was linked to depressive symptomatology in elderly Swedish 1 year after stroke (41). Chatterjee et al. found that stroke patients with higher Hcy and lower folate levels may be more susceptible to PSD (42). A meta-analysis reported that the high Hcy level in the acute phase of ischemic stroke may be a risk factor for PSD (43). These are in accordance with our results, and we further revealed that Hcy was regulated by MTHFR rs1801133 to affect PSD.

The association between Hcy and PSD might be explained by several potential pathophysiological pathways. Firstly, Hcy may influence depressive symptoms *via* alterations of neurotransmitters. Previous research showed that depressed patients with raised Hcy level had decreased serum, red cell, and cerebrospinal fluid folate and cerebrospinal fluid S-adenosylmethionine (44). S-adenosylmethionine not only has antidepressant properties but also acts as the methyl donor involved in methylation reactions of monoamines and neurotransmitters (45–47). Hcy treatment significantly decreased central monoamines levels (norepinephrine, dopamine, and 5-hydroxytryptamine) in post-stroke rats (48). Secondly, Hcy is a toxic substance and can cause damage to the central nervous system. Hcy treatment aggravated depressive-like disorders and contributed to the decrease in the number of synapses and postsynaptic density in post-stroke rats, compared to the control group (48). Thirdly, a strong association between Hcy level and inflammation has been reported in various studies (49–51), and inflammatory processes have been implicated in the pathophysiology of depression (52).

Some limitations of this study should be noted. Firstly, it was an observational study with a relatively small sample size, and patients were only recruited in one clinical institution. The conclusion could not be over-interpreted. Secondly, we excluded those patients with a severe condition and severe aphasia, which would result in biases for the prevalence of PSD. Thirdly, the Hcy level of patients was measured at admission, and the intervals between measurement and stroke onset were different. Fourthly, we did not record the history of taking vitamins B or folic acid. Future researchers need to consider the effect of vitamins B and folate on Hcy level. Fifthly, we only focused on Hcy at admission and PSD at 2 weeks after stroke. Further studies with long-term follow-up samples are required to explore how Hcy levels change during the course of PSD.

In conclusion, our study suggested that Hcy and rs1801133 AA genotype, A allele, and haplotype GTAG (rs11559040-rs1801131-rs1801133-rs2066462) may be associated with the increased risk of early-onset PSD. The rs1801133 may affect early-onset PSD *via* mediating Hcy level. These results can shed new light on the mechanism of PSD. Clinicians should pay attention to the Hcy level of ischemic stroke patients and evaluate depressive symptoms timely. Hcy-lowering treatment may be a potential therapeutic target for the prevention and intervention of PSD.

## Data availability statement

The original contributions presented in this study are included in the article/Supplementary material, further inquiries can be directed to the corresponding authors.

## Ethics statement

The study involving human participants were reviewed and approved by the Ethics Committee of Xiangya Hospital of Central South University (No. 201910842). The patients/participants provided their written informed consent to participate in this study.

## Author contributions

FT and MS conceived and designed the protocol. JZ, HC, FL, QL, DS, YX, WX, and DZ recruited and evaluated patients. CZ and XH analyzed the data. JZ interpreted the results and wrote the manuscript. All authors contributed to the article and approved the submitted version.

## Footnotes

1
http://www.ncbi.nlm.nih.gov/SNP

2
http://analysis.bio-x.cn/
