# Supplementary material for: Association of homocysteine and polymorphism of methylenetetrahydrofolate reductase with early-onset post stroke depression
Source: Front Nutr. 2022 Dec 6;9:1078281. doi: 10.3389/fnut.2022.1078281 (PMC9763289; doi:10.3389/fnut.2022.1078281)
Supplement: Supplementary file 1 [file Table_1.DOCX]

Supplementary Table 1: Association of genotypes and alleles of MTRR and MTR and PSD

| Genotypes and Alleles | PSD | Non-PSD | OR [CI_95%_] | *p*-value | Adjusted OR [CI_95%_] | *p*-value |
| --- | --- | --- | --- | --- | --- | --- |
| rs10380 (MTRR) |  |  |  |  |  |  |
| C/C | 54 | 87 | Ref | - | Ref | - |
| C/T | 23 | 42 | 0.882 [0.479~1.626] | 0.688 | 1.043 [0.544~1.999] | 0.900 |
| T/T | 4 | 2 | 3.222 [0.571~18.194] | 0.185 | 3.509 [0.565~21.773] | 0.178 |
| C | 131 | 216 | 0.900 [0.543~1.490] | 0.682 | 0.800 [0.473~1.354] | 0.406 |
| T | 31 | 46 |  |  |  |  |
| rs1532268 (MTRR) |  |  |  |  |  |  |
| C/C | 56 | 101 | Ref | - | Ref | - |
| C/T | 24 | 28 | 1.546 [0.819~2.919] | 0.179 | 1.253 [0.637~2.464] | 0.513 |
| T/T | 1 | 2 | 0.902 [0.080~10.167] | 0.933 | 0.281 [0.021~3.667] | 0.333 |
| C | 136 | 230 | 0.728 [0.416~1.273] | 0.265 | 0.956 [0.526~1.736] | 0.882 |
| T | 26 | 32 |  |  |  |  |
| rs161870 (MTRR) |  |  |  |  |  |  |
| T/T | 51 | 77 | Ref | - | Ref | - |
| C/T | 26 | 50 | 0.785 [0.435~1.418] | 0.423 | 0.956 [0.511~1.788] | 0.888 |
| C/C | 4 | 4 | 1.510 [0.361~6.311] | 0.572 | 1.672 [0.371~7.543] | 0.504 |
| T | 128 | 204 | 0.934 [0.580~1.506] | 0.780 | 1.069 [0.650~1.758] | 0.793 |
| C | 34 | 58 |  |  |  |  |
| rs1801394 (MTRR) |  |  |  |  |  |  |
| A/A | 41 | 81 | Ref | - | Ref | - |
| G/A | 34 | 45 | 1.493 [0.834~2.673] | 0.178 | 1.331 [0.719~2.463] | 0.363 |
| G/G | 6 | 5 | 2.371 [0.683~8.232] | 0.174 | 1.995 [0.555~7.172] | 0.290 |
| A | 116 | 207 | 0.670 [0.426~1.054] | 0.083 | 0.741 [0.461~1.190] | 0.215 |
| G | 46 | 55 |  |  |  |  |
| rs2287779 (MTRR) |  |  |  |  |  |  |
| G/G | 59 | 90 | Ref | - | Ref | - |
| G/A | 19 | 39 | 0.743 [0.392~1.408] | 0.363 | 0.657 [0.334~1.290] | 0.222 |
| A/A | 3 | 2 | 2.288 [0.371~14.109] | 0.372 | 1.711 [0.249~11.767] | 0.585 |
| G | 137 | 219 | 0.929 [0.543~1.590] | 0.789 | 0.819 [0.467~1.437] | 0.487 |
| A | 25 | 43 |  |  |  |  |
| rs12070777 (MTR) |  |  |  |  |  |  |
| C/C | 33 | 43 | Ref | - | Ref | - |
| A/C | 35 | 64 | 0.713 [0.386~1.315] | 0.278 | 0.703 [0.368~1.343] | 0.286 |
| A/A | 13 | 24 | 0.706 [0.313~1.592] | 0.401 | 0.731 [0.313~1.704] | 0.468 |
| C | 101 | 150 | 0.809 [0.542~1.208] | 0.300 | 0.821 [0.540~1.248] | 0.355 |
| A | 61 | 112 |  |  |  |  |
| rs1805087 (MTR) |  |  |  |  |  |  |
| A/A | 64 | 100 | 1.167 [0.597~2.280] | 0.651 | 1.093 [0.544~2.193] | 0.803 |
| A/G | 17 | 31 |  |  |  |  |
| A | 145 | 231 | 1.145 [0.612~2.143] | 0.673 | 1.080 [0.564~2.071] | 0.816 |
| G | 17 | 31 |  |  |  |  |
| rs3738547 (MTR) |  |  |  |  |  |  |
| C/C | 71 | 117 | 0.850 [0.358~2.015] | 0.711 | 0.953 [0.379~2.394] | 0.919 |
| C/T | 10 | 14 |  |  |  |  |
| C | 152 | 248 | 0.858 [0.372~1.980] | 0.720 | 0.956 [0.392~2.333] | 0.921 |
| T | 10 | 14 |  |  |  |  |

**Abbreviations:** SNP, single nucleotide polymorphism; PSD, post stroke depression; OR, odds ratio; CI, confidence interval; MTR, methionine synthase; MTRR, methionine synthase reductase.

**Adjusted model:** Adjusted for age, gender, body mass index, NIHSS score, MMSE score.
